# Incidental Irregular Mass Within a Hamartoma Detected on Breast Ultrasound: A Case Report and Review of the Literature

**DOI:** 10.7759/cureus.102641

**Published:** 2026-01-30

**Authors:** Christine Tang, Justin H Qian, Miguel A Salinas, Kellen S Carril, Quan D Nguyen

**Affiliations:** 1 Radiology, Baylor College of Medicine, Houston, USA; 2 Psychiatry, University of Maryland School of Medicine, Baltimore, USA; 3 Radiology, University of Texas Medical Branch, Houston, USA

**Keywords:** bi-rads 4 lesion, breast carcinoma, breast ultrasound, fibroadenoma, mammary hamartoma, mammogram

## Abstract

Mammary hamartomas are heterogeneous breast masses containing glandular epithelium, fibrous tissue, and adipose tissue. The development of a breast carcinoma within a hamartoma is rare. We present the case of a 41-year-old female patient who had an asymptomatic breast mass incidentally discovered on physical exam. Ultrasound indicated a benign-appearing mass encased in a hamartoma. Fifteen months later, follow-up imaging detected a new hypoechoic irregular mass with an angular margin. Further work-up with biopsy identified the mass to be a fibroadenoma. In hypoechoic masses with suspicious features such as irregular shape and angular margins, malignancy cannot be definitively ruled out without a core needle biopsy. Radiological identification of these masses, which appear distinct from heterogeneous hamartomas, allows for early detection of potential carcinomas. To distinguish carcinomas from fibroadenomas, these masses should be biopsied.

## Introduction

Hamartomas are circumscribed masses consisting of multiple tissues from the surrounding organ. Breast hamartomas consist of adipose tissue, fibrous tissue, and glandular epithelium [1]. They grow slowly and can be as large as 12 cm in diameter. However, they may also be small and undetected by palpation. When detected on a physical exam, hamartomas manifest as soft, round, smooth, and mobile masses without associated symptoms.

On ultrasound, hamartomas appear as well-defined, solid, oval masses, containing a mixture of hypoechoic areas and hyperechoic nodular or band-like areas [1]. This heterogeneous mixture represents the combination of tissue types in a hamartoma.

On mammography, hamartomas similarly present as oval areas containing a mixture of radiotransparent and radio-opaque areas [1]. A characteristic mammographic finding is a thin, radio-opaque pseudocapsule.

Once diagnosed, mammary hamartomas are considered benign and typically do not require excision [2]. Hamartomas are not considered a risk factor for the development of breast malignancies. However, because hamartomas contain glandular tissue, malignant transformation is possible. Therefore, malignancy is a possible differential when a suspicious mass is discovered in association with a hamartoma.

In a 2010 review of the literature, Choi and Ko found 15 cases of hamartoma-associated malignancy [2]. Etiologies included lobular carcinoma in situ, ductal carcinoma in situ, invasive lobular carcinoma, and invasive ductal carcinoma. Ultrasound was completed in six cases, four of which contained suspicious features such as hypoechogenicity, irregular margins, and non-parallel orientation. In some cases of hamartoma-associated malignancy, mammography also revealed notable features such as irregular margins, focal asymmetry, spiculation, and microcalcifications in the carcinomas. The carcinomas ranged in size from 0.3 to 3.5 cm in diameter. 

In addition to malignancies, one of the most common etiologies of a breast mass is the fibroadenoma. A fibroadenoma is a benign tumor consisting of epithelial and stromal tissues in the breast [3]. These tissues’ sensitivity to estrogen results in the development of fibroadenomas. For this reason, risk factors include mechanisms leading to unopposed estrogen, such as pregnancy and oral contraceptive pill use. Fibroadenomas are most common during the ages of 14-35, due to estrogen exposure, and uncommon after menopause.

Upon initial clinical presentation on physical exam, fibroadenomas usually manifest unilaterally in the upper outer quadrant as a painless, non-tender, solid, and mobile mass [3]. The mass typically has a rubbery consistency and solid borders.

To confirm the diagnosis of a fibroadenoma after clinical evaluation, imaging with mammograms and ultrasounds is important. Mammography is the imaging modality of choice for masses in women above the age of 35. On mammography, fibroadenomas usually appear as a distinct area with smooth, round edges [3]. Many cases appear as a discrete round hypodense or isodense mass, although some cases may involve macrolobulated or partially obscured margins. In older patients, especially postmenopausal ones, fibroadenomas may contain calcifications that produce the classic coarse popcorn calcification appearance.

The Breast Imaging Reporting and Data System (BI-RADS) is a standardized system of breast pathology reporting for mammograms, ultrasounds, and magnetic resonance imaging. This system was developed by the American College of Radiology, with the most recent 5^th^ edition released in 2013. Imaging studies are assigned one of seven assessment categories to indicate the level of suspicion for malignancy (BI-RADS 0-6) and a series of standardized terminology to describe breast imaging findings among mammography, ultrasound, and MRI [4].

Breast ultrasound is superior for detecting fibroadenomas in women younger than age 35. In this population, ultrasonography is useful for detecting abnormalities within dense breast tissue. Similar to mammography, ultrasonography of fibroadenomas presents as well-circumscribed masses that are hypoechogenic throughout [5]. The shape of the mass may be round, ovoid, or macrolobulated.

If a fibroadenoma cannot be clearly diagnosed on imaging, a core needle biopsy is recommended for a histopathological workup [6]. Once a fibroadenoma is diagnosed, typical management is conservative. Exceptions warranting surgical removal include rapid growth, size greater than 2 cm, and patient request [3]. 

## Case presentation

**Figure 1:**
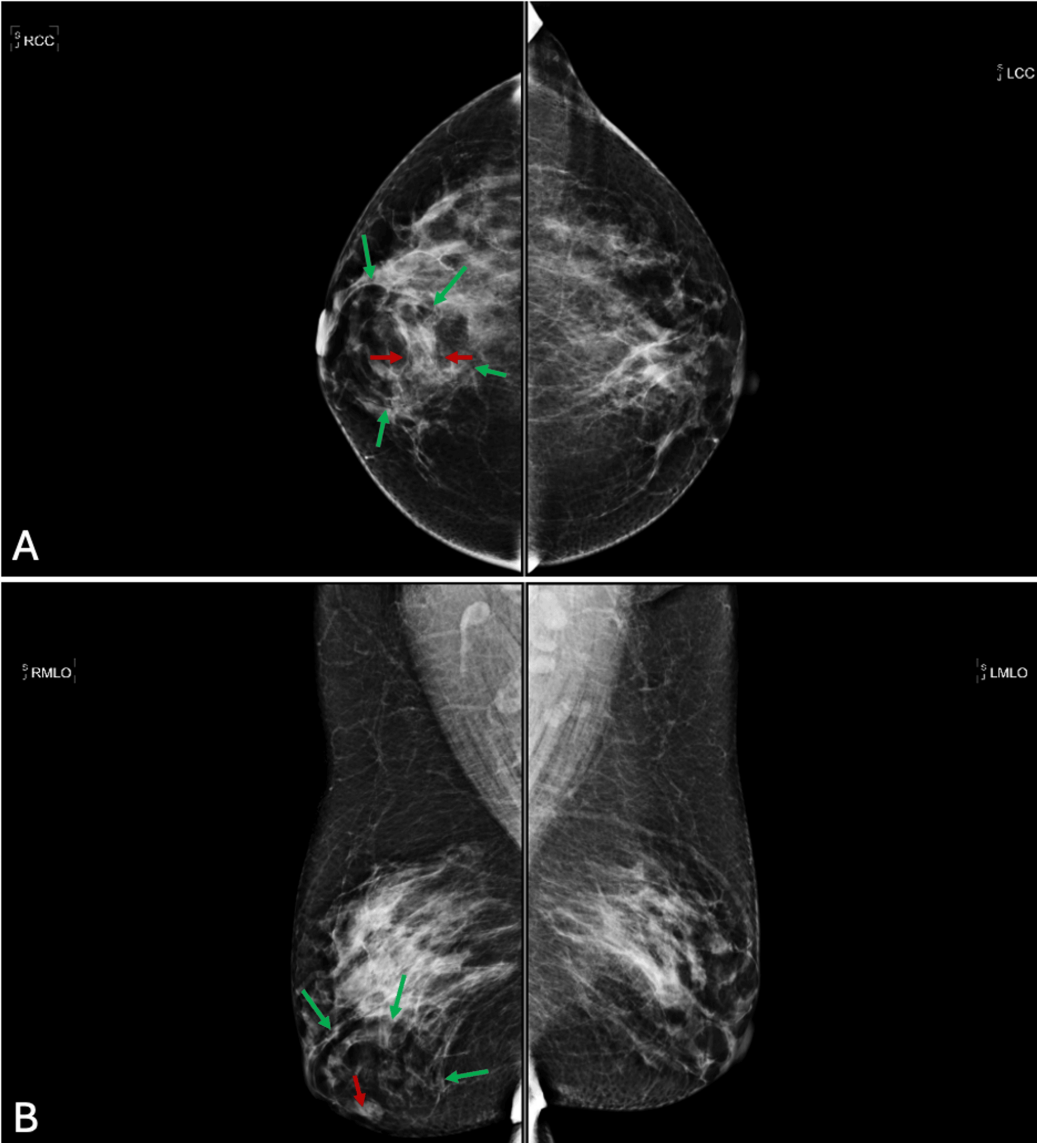
Screening mammogram Mammography of the right breast mass at 5 o’clock. A. Craniocaudal view; initial screening mammography showed a 1.4 cm indeterminate focal asymmetry in the anterior right breast. B. Mediolateral oblique view of the initial mammogram; the green arrows depict the hamartoma, and the red arrow depicts the indeterminate focal asymmetry.

A 41-year-old female patient presented to the mammography clinic for evaluation of a right breast mass. The mammogram showed a 1.4 cm indeterminate focal asymmetry (Figures 1A, 1B).

**Figure 2:**
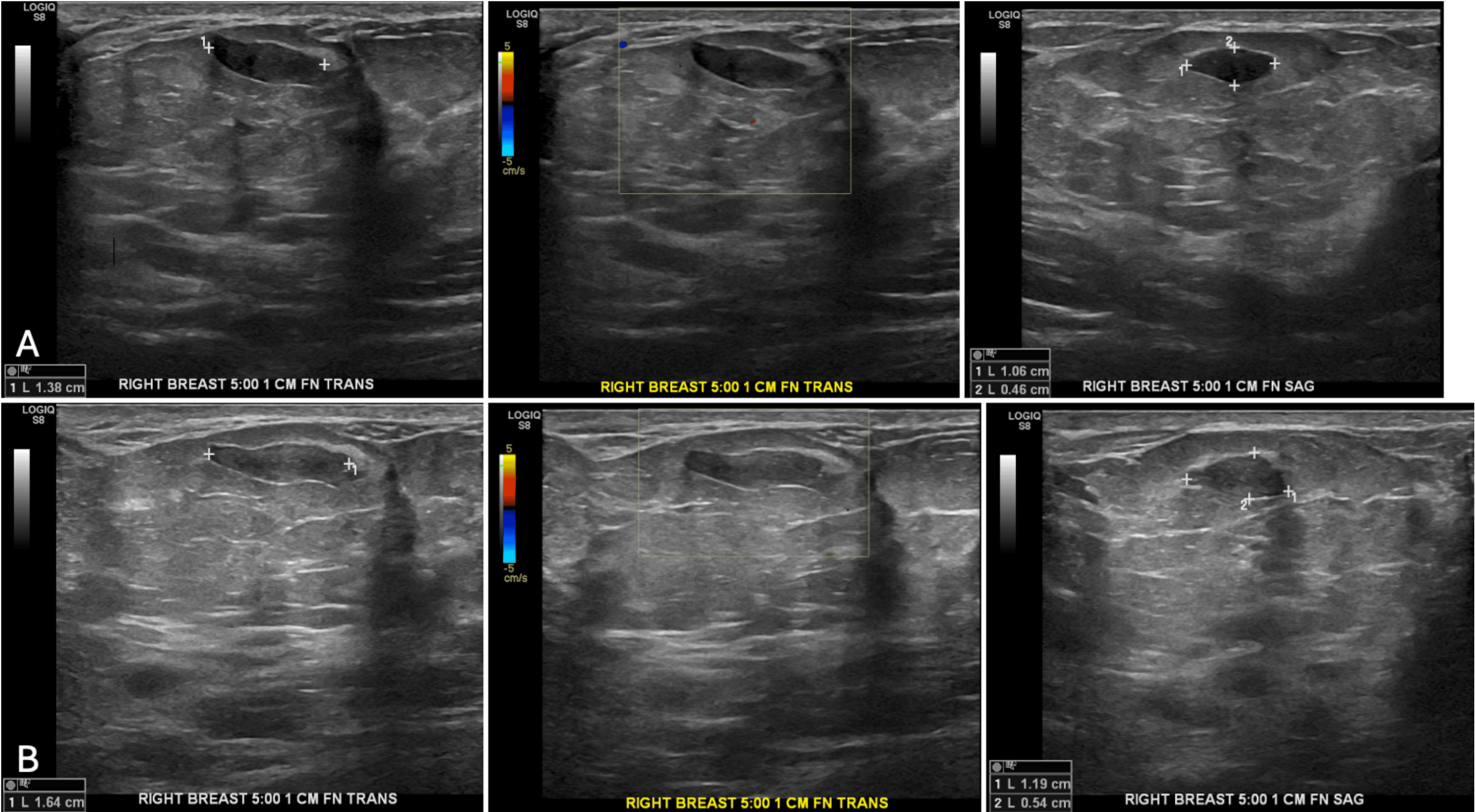
Ultrasonography of the breast mass in the right breast at 5 o'clock A. Two months after the initial mammogram, a follow-up ultrasound demonstrated a 1.4 x 0.5 x 1.1 cm oval mass with circumscribed margins within a hamartoma. B. Nine months after the initial mammography, the same oval mass was grossly stable and measured 1.6 cm x 0.5 cm x 1.2 cm.

Two months later, follow-up ultrasound revealed a 1.4 cm x 0.5 cm x 1.1 cm oval mass with a circumscribed margin in the right breast at 5 o'clock, anterior depth 1 cm from the nipple, encased in a benign hamartoma. Nine months after initial mammography, ultrasound revealed a 1.6 cm x 0.5 cm x 1.2 cm hypoechoic oval mass with a circumscribed margin at the same location (Figures 2A, 2B).

**Figure 3:**
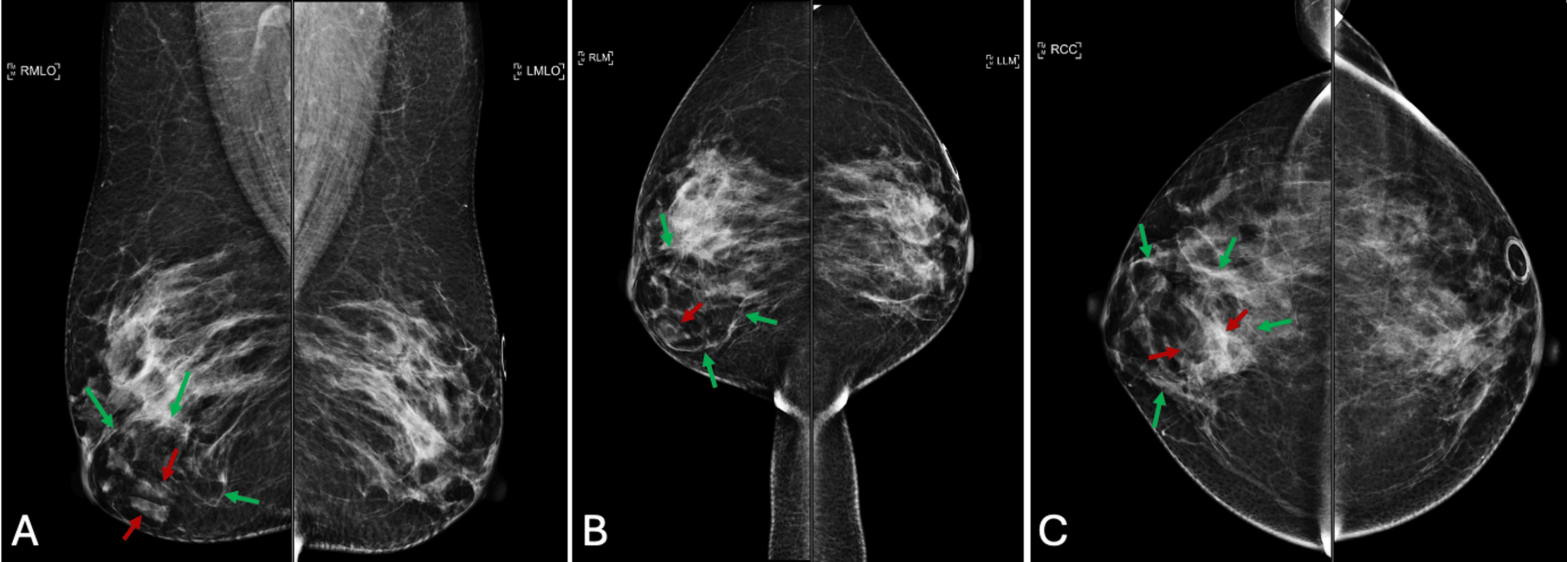
A follow-up mammogram at 15 months after the initial mammogram A. Mediolateral oblique view; a follow-up mammogram demonstrated that the mass was stable and likely benign. The green arrows depict the hamartoma, and the red arrows depict the indeterminate mass. B. Lateromedial view of the follow-up mammogram. C. Craniocaudal view.

Fifteen months after initial mammography, a follow-up mammogram demonstrated that the previous mass was stable (Figures 3A-3C).

**Figure 4:**
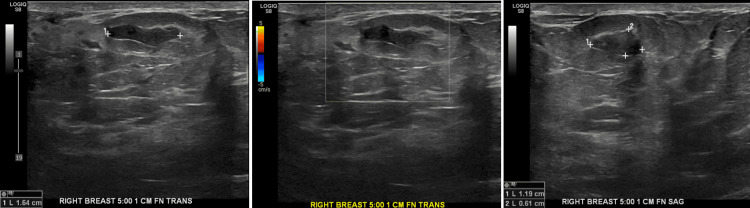
Follow-up ultrasonography at 15 months after initial mammography Ultrasonography of the breast mass in the right breast at 5 o'clock. The same oval mass was stable at 1.6 x 0.6 x 1.2 cm.

The follow-up ultrasound showed the hypoechoic oval mass to be stable (Figure 4).

**Figure 5:**
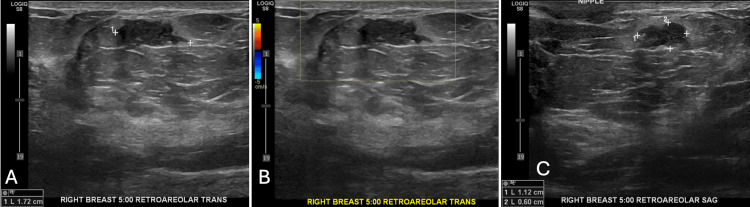
An incidental mass was found on the ultrasound 15 months after the initial screening. Follow-up ultrasound incidentally demonstrated a separate 1.7 cm irregular mass with an angular margin in the retroareolar region. This had intermediate suspicion for malignancy. A. Transverse view. B. The mass had no vascularity on color Doppler ultrasound. C. Sagittal view.

However, this ultrasound also incidentally revealed a new 1.7 cm x 0.6 cm x 1.1 cm irregular mass with an angular margin in the right breast at 5 o'clock in the retroareolar region (Figures 5A-5C).

Due to intermediate risk of malignancy (BI-RADS 4b), the incidentally found irregular mass was biopsied. Biopsy determined the irregular mass to be a fibroadenoma. She was scheduled for follow-up in one year of diagnosis with a mammogram and a right breast ultrasound.

## Discussion

Fibroadenomas and phyllodes tumors are classified as fibroepithelial lesions (FELs) that demonstrate proliferation of both epithelial and stromal cells [7]. The fibroadenoma is the most commonly diagnosed benign solid mass, while phyllodes tumors are rarer and are classified as benign, borderline, and malignant. Fibroadenomas typically present on ultrasound as round or oval, well-circumscribed masses. However, approximately 25% of fibroadenomas present as an irregular hypoechoic mass, as in this case [8]. Irregular hypoechoic masses indicate suspicion for malignancy. Fibroadenomas may have posterior shadow or enhancement on a gray-scale image, as well as peripheral feeding vessels on color Doppler ultrasound [9].

Irregular hypoechoic masses can be classified into BI-RADS categories 4A (low suspicion), 4B (moderate suspicion), 4C (high suspicion), or 5 (highly suggestive of malignancy) on ultrasound. Among BI-RADS category 4 lesions, fibroadenomas have been found to be the most common histological differential [10]. Thirty-eight percent of these imaging manifestations were classified as fibroadenomas on follow-up biopsy. Other differential diagnoses include sclerosing adenosis (18% of cases), fibrocystic changes (14%), and mastitis (9.5%).

For BI-RADS category 4 lesions, the recommended next step in management is to biopsy the mass [11]. Once biopsied, histopathology differentiates malignancies from benign etiologies. BI-RADS subcategories 4A, 4B, and 4C have been found to have a positive predictive value of 9%, 21%, and 57%, respectively, for malignancy.

Clinically, fibroadenomas are asymptomatic in 25% of women, and when symptomatic, they usually present in 20- to 30-year-old females as a single, firm, mobile mass. Fibroadenomas can be clinically observed safely but may be excised due to breast pain and/or patient preference after clinician-patient discussion [7].

Hamartomas of the breast are rare and benign, and they are usually diagnosed incidentally. They consist of glandular and epithelial elements and fibrous and adipose tissue [12]. Ultrasound features include a well-circumscribed, oval solid mass with heterogeneous echogenicity [13]. In addition, breast carcinomas may arise from or near a benign hamartoma [2]. However, this is rare, as only 16 cases have been reported in the literature [13]. Radiologists should be vigilant of hamartoma-associated masses with suspicious imaging features, such as irregular margins, spiculation, and non-parallel orientation on ultrasonography or mammography. Hamartomas may be surgically resected, which is the definitive treatment [12, 14]. There are currently no definite treatment or adjuvant therapy guidelines for hamartomas that have converted to carcinoma [14]. Although this patient had a benign fibroadenoma, it was important to rule out malignancy with a core needle biopsy, as the retroareolar mass was new and found incidentally on ultrasound with imaging features that showed an intermediate suspicion for malignancy.

To our knowledge, this is the first reported case of hamartoma-associated fibroadenoma in the literature. However, considering the prevalence of fibroadenoma among BI-RADS category 4 masses, we would expect fibroadenoma to be a relatively frequent finding in the workup of suspicious hamartoma-associated masses. 

## Conclusions

Hamartomas are large, benign, heterogeneous masses containing epithelial tissue, which rarely give rise to carcinomas. On ultrasound and mammogram, carcinomas may appear within a hamartoma as hypoechoic masses with suspicious features, such as irregular and angular margins. Radiologists play a key role in identifying suspicious masses from the surrounding hamartoma. Early detection allows for prompt core needle biopsy, which can distinguish carcinomas from benign etiologies such as fibroadenomas.
